# Addressing Critiques of the Evidence Linking Fluoride and Children’s IQ

**DOI:** 10.5334/aogh.4853

**Published:** 2025-12-12

**Authors:** Kyla W. Taylor, Sorina E. Eftim, Christopher A. Sibrizzi, Robyn B. Blain, Kristen Magnuson, Pamela A. Hartman, John R. Bucher, Andrew A. Rooney

**Affiliations:** 1 Division of Translational Toxicology, National Institute of Environmental Health Sciences, National Institutes of Health, Research Triangle Park, NC, USA; 2 ICF, Reston, VA, USA

**Keywords:** fluoride, neurodevelopment, IQ, systematic review, meta-analysis

## Abstract

We recently completed a comprehensive systematic review of the literature on fluoride exposure and neurodevelopment and cognition, resulting in two publications. The 2024 National Toxicology Program Monograph concluded—with *moderate confidence*—that higher fluoride exposure is associated with lower IQ in children. The 2025 meta‑analysis, published in *JAMA Pediatrics*, quantitatively synthesized over 70 epidemiological studies and likewise reported an inverse association between fluoride exposure and children’s IQ. This inverse association persisted when analyses were restricted to the best evidence, the high‑quality studies, and was consistent across subgroups defined by sex, age, country, outcome assessment method, timing of exposure, and exposure matrix (e.g., urine or drinking water). Notably, among the high‑quality evidence, inverse associations were still observed at fluoride exposure levels below 1.5 mg/L, based on both urinary and drinking‑water measurements. These publications have received considerable public and media attention, prompted healthy scientific discourse, and have been cited by public health decision‑makers. Many scientific comments were carefully considered and resolved during development and peer review, which contributed to the rigor of the final documents. However, some recurrent critiques continue to be raised. This viewpoint provides a high‑level summary of these key critiques and corresponding responses to help the public, media, and the scientific community better understand the strength and implications of the scientific evidence on fluoride exposures and neurodevelopment and cognition.

## Introduction

Evolving evidence has renewed scientific interest and public debate over safe limits for fluoride in drinking water. In April 2025, the US Department of Health and Human Services announced it was reconvening the Community Preventive Services Task Force “to study and make a new recommendation on fluoride” [1] and the US Environmental Protection Agency (EPA) announced it would review the scientific evidence surrounding fluoride in drinking water and its potential health risks [2].

These recent developments mark the latest chapter in a long‑standing debate over potential neurological effects of fluoride. In 2006, the National Research Council (NRC) concluded that high levels of naturally occurring fluoride in drinking water may pose risks to cognition and behavior [3]. Although the NRC only cited five epidemiological studies, they noted that the evidence warranted further research on fluoride’s potential effects on intelligence.

In July 2016, the National Toxicology Program (NTP) published a systematic review of the experimental animal evidence, which concluded there was *low‑to‑moderate evidence* that fluoride exposure has adverse effects on learning and memory in animals [4].

Also in 2017, the Division of Translational Toxicology (DTT) at the National Institute of Environmental Health Sciences (NIEHS) initiated an expanded systematic review to include experimental animal, mechanistic, and human epidemiological evidence of effects of fluoride on neurodevelopment and cognition. The human data provided the strongest evidence, and because most of the high‑quality epidemiological studies focused on IQ in children, they formed the primary basis for the conclusions. The systematic review, published in August 2024 as an NTP Monograph, concluded—with *moderate confidence*—that higher fluoride exposure is associated with lower IQ in children [5].

By the end of 2024, the number of epidemiological studies examining this association had increased several‑fold since the 2006 NRC report, to include over 70 studies conducted across 12 countries, utilizing diverse study designs, and 22 high‑quality studies. In January 2025, the DTT authors published a detailed meta‑analysis of these 74 epidemiological studies in *JAMA Pediatrics,* reporting an inverse association between fluoride exposure and children’s IQ scores [6].

A principal finding of the meta‑analysis [6] was a statistically significant inverse association between fluoride exposure and children’s IQ scores (β = 1.63; 95% CI: −2.33 to −0.93; p < 0.001). The inverse association held when restricted to only high‑quality studies and across subgroups based on sex, age, country, outcome assessment type, timing of exposure, and exposure matrix (e.g., urine or drinking water). Notably, among the best available evidence (i.e., the high‑quality studies), inverse associations were observed when fluoride exposure was restricted to less than 1.5 mg/L as estimated by measurements in both urine and drinking water.

The publication of the NTP Monograph and meta‑analysis followed a rigorous and unprecedented peer review process, which included evaluation by a National Academy of Sciences, Engineering and Medicine (NASEM) committee. The NTP Monograph then underwent the established NTP peer review process (i.e., review by five external experts in neurodevelopment and cognition). Additional reviews of both documents were conducted by staff from other NIH institutes and the Centers for Disease Control. Finally, the authors’ responses to comments from each round of review were further reviewed by an ad hoc group of subject‑matter experts convened by the NTP Board of Scientific Counselors. Both DTT publications [5, 6] garnered extensive comments throughout their development and the various peer reviews. Specific comments from these reviews, along with responses, are available on the NTP website [7]. The meta‑analysis underwent additional peer review overseen by editors at *JAMA Pediatrics.*

During the same time period, there was legal action focusing on the risks of fluoride exposure. In 2017, citing concerns about potential neurotoxicity, several nonprofit organizations and individuals filed a federal lawsuit under the Toxic Substances Control Act (TSCA) regarding health impacts of water fluoridation [8]. That lawsuit, *Food and Water Watch et al. v. US EPA* [9], was heard in US District Court beginning in June 2020 and proceedings were paused pending peer review and publication of the NTP Monograph and meta‑analysis. The trial resumed in January 2024 using a pre‑publication version of the monograph and a draft of the meta‑analysis, both of which had been requested by the court and, along with existing peer reviews, were made publicly available prior to publication. In September 2024, the court ruled that water fluoridation at the US Public Health Service’s recommended level of 0.7 mg/L posed an unreasonable risk of reducing IQ in children and ordered EPA to take action under TSCA, at minimum, to formally review the scientific evidence for neurotoxicity [9].

Both the NTP Monograph and the meta‑analysis have been subject to careful peer review prior to publication, during which comments and critiques were raised and addressed. Given the strong ongoing interest in the topic, we developed a high‑level summary of key critiques that is outlined below along with bulleted responses to assist those reviewing the current scientific evidence related to fluoride exposures and neurodevelopment and cognition.

## Key Critiques and Authors’ Responses

### Uncertainty from low‑quality studies

Several commenters suggested that including low‑quality studies biased the conclusions.

- Best practice in systematic reviews (e.g., recommended by Cochrane) is to include all studies, apply pre‑established risk‑of‑bias criteria, provide transparent rationale for risk‑of‑bias ratings, and analyze subgroups based on study quality. Excluding studies without subgroup analysis can introduce bias, reduce power, and compromise the integrity of the review.
- In Taylor et al., analyses restricted to high‑quality studies—which represent the best, most reliable evidence—consistently supported and affirmed the inverse association between fluoride exposure and children’s IQ.

### Exclusion of cross‑sectional studies

Some commenters proposed that cross‑sectional studies should be excluded based on a misconception that they cannot provide evidence that exposure precedes (and therefore could cause) the outcome.

- Although cross‑sectional studies typically assess exposure and outcome simultaneously, many in the fluoride literature restricted analyses to children who lived in high fluoride areas since birth or showed signs of long‑term exposure (e.g., dental fluorosis) and therefore provide evidence that exposure to fluoride preceded measurement of IQ [5].
- As noted by Savitz [10], excluding studies based on study design label (e.g., cross‑sectional) can introduce bias and discard valuable evidence.
- The monograph and Taylor et al. followed best practices to systematically and rigorously evaluate each study individually and transparently presented findings from all included studies.

### Assumption that any heterogeneity is problematic

Some commenters suggested that any form of heterogeneity is problematic.

- Heterogeneity, or variability, is expected and appropriate in observational studies which often differ by study populations, study locations, exposure levels, or outcome measures [11–13].
- It is important to distinguish between the two types of heterogeneity:
  - (1) *Heterogeneity in results* refers to inconsistent findings across independent studies evaluating the same endpoint that cannot be explained by study design or methodological features and may reduce confidence in an overall association. In systematic review of observational studies, this is assessed primarily by evaluating whether the direction of associations is consistent across studies [11–13]. (Note: heterogeneity in results was not observed in the Taylor et al. analyses; 19 of the 22 high‑quality studies reported inverse associations.)
  - (2) *Heterogeneity in methods* refers to differences in study design, populations, methods, or study quality and can strengthen confidence in an association if the direction of association remains consistent across studies [11–13]. In meta‑analysis, this type of heterogeneity can be measured by the I^2^ statistic, the percentage of variation across studies due to differences between studies, rather than chance [12]. (Note: the body of evidence in Taylor et al. had moderate to high heterogeneity in the regression coefficients and mean‑effects analyses [I^2^= 60% and 94%, respectively].)
- In Taylor et al., the inverse association between fluoride and IQ remained consistent despite differences in methods and study populations, supporting the robustness of the overall associations.

### Validity of urinary measurements

Some comments disagreed with the validity of urinary fluoride measurements as a reliable exposure estimate.

- This critique does not reflect the scientific consensus (e.g., regulatory organizations like the EPA routinely rely on urinary measurements as exposure estimates in risk assessments).
- Moreover, the inverse associations between fluoride exposure and children’s IQ were consistently observed across multiple exposure metrics including drinking water and estimated fluoride intake—not just urinary fluoride.
- This consistency of associations relative to different exposure metrics (estimated intake, drinking water, and urinary measures) strengthens the validity of the observed associations, and indicates that the associations are not driven by any single exposure measurement method.

### Timing of exposure

It has been noted that Taylor et al. did not include discussion of prenatal exposure vs. postnatal exposure.

- There were three high‑quality prospective cohort studies that combined repeated urinary measurements over the course of pregnancy to examine prenatal fluoride exposure during this critical period of brain development.
- Compared to the pooled effect estimate for postnatal fluoride exposure and children’s IQ (β = −1.65; 95% CI: −2.39 to −0.93), the pooled effect estimate for prenatal fluoride exposure (as measured by maternal urinary fluoride) was larger, but not statistically significant (β = −1.70; 95% CI: −4.23 to 0.84). These data are available in the supplemental material of Taylor et al.
- Additional prospective cohort studies with repeated prenatal fluoride measurements would provide valuable evidence to strengthen this important area of research [14].

### Exposure misclassification

Some commenters suggested that measuring fluoride exposure via urinary biomarkers introduces systematic exposure misclassification that biases associations *away from the null* or overestimates the relationship between fluoride exposure and children’s IQ.

- For exposure misclassification to bias associations away from the null, it would have to occur *differentially* by outcome (i.e., measurement errors would have to be related to select outcomes) across the entire evidence base. For example, it would not be sufficient for spot urine samples to misclassify all children as having lower exposure; to produce bias away from the null, the misclassification would have to selectively underestimate fluoride exposure specifically among children who have both high fluoride exposure and lower IQ scores.
- *Non‑differential* misclassification (where errors occur independently of select outcomes) introduces random noise into the data and typically biases observed associations *towards the null* and tends to underestimate the true association.
- When statistically significant associations are observed despite non‑differential misclassification, as was found in Taylor et al., the true association may in fact be stronger than what is observed.

### Confounding

Some commenters have said that the assessment of confounding in individual studies was insufficient or inconsistent.

- In Taylor et al., confounding was consistently assessed in all studies as a primary risk‑of‑bias domain using pre‑specified methods described in a peer‑reviewed protocol.
- Following the protocol, studies were only rated low risk of bias for confounding if they adequately accounted for key covariates such as age, sex, socioeconomic status, and co‑exposures to other neurotoxicants (e.g., lead, arsenic).
- Additional important covariates (e.g., race/ethnicity, smoking, parental education) were also considered based on study population context.
- Most high‑quality studies (the best evidence) did not have issues with confounding; 16 of 19 studies in the monograph and 17 of 22 studies in Taylor et al. were rated low risk of bias for confounding.
- Sensitivity analyses showed that confounding could not explain the observed inverse associations between fluoride exposure and children’s IQ across the body of evidence [5].

### Clustering

Clustering occurs in studies where participants are not truly independent of each other because they are grouped or “clustered” in some way, potentially leading to underestimated standard errors or biased effect estimates. Some commenters suggested that this was not considered in the DTT systematic reviews.

- In response to early feedback from NASEM, we added a specific consideration of clustering to the monograph and Taylor et al.
- All high‑quality studies were re‑evaluated for potential bias related to clustering.
- For studies where clustering concerns were applicable, we revised the risk of bias assessment to address them.
- Further details can be found in the supplemental material of Taylor et al.

### Publication bias

Publication bias occurs when studies with certain results are more likely to be published. Some commenters suggested that Taylor et al. overlooked recent negative studies and meta‑analyses.

- The inclusion and exclusion of studies were determined by pre‑specified criteria in the protocol to select studies relevant to fluoride exposure and children’s IQ.
- Taylor et al. did not include any study that did not meet inclusion criteria (e.g., did not evaluate IQ) or was not published in the peer‑reviewed literature. Specific studies are discussed in the Taylor et al. supplemental material.
- Best practices in meta‑analyses involve evaluating publication bias with tests like Egger’s and Begg’s, funnel plots, and trim‑and‑fill analyses, and by presenting results transparently. Taylor et al. conducted these analyses and concluded that publication bias could not account for the findings.

### Non‑Medline indexed journals

Some commenters suggested that publications from non‑Medline indexed journals should not have been included.

- Consistent with best practices for assessing study quality (e.g., Cochrane), the journal of publication is not considered a valid or reliable indicator of study quality.
- The monograph and Taylor et al. followed best practices to systematically and rigorously evaluate each study individually and to transparently present findings from all included studies.

### Transparency

Some commenters suggested a lack of transparency in these analyses.

- Comprehensive materials were publicly posted to the NTP website prior to journal submission including protocol and revisions, pre‑submission manuscript drafts, NASEM reviews, internal and interagency reviews, written responses to all aforementioned reviews, and subsequent independent NTP BSC review of those responses.
- To promote transparency, full public access to included studies (study details and extracted data), the list of excluded studies (with justifications), and risk‑of‑bias ratings and rationales for each rating are provided at https://hawcproject.org/assessment/405/.

### Overlapping data

Some commenters have said that Taylor et al. included overlapping data from multiple publications based on the same Tianjin, China cohort.

- Taylor et al. followed established guidelines to ensure that data from the same study populations were not included multiple times in analyses.
- Further details regarding the distinctions between these studies are provided in the supplementary material.

## Conclusions

Confidence in the inverse association between fluoride exposure and children’s IQ, as reported in the 2024 systematic review [5] and 2025 meta‑analysis [6], is based on robust evidence from numerous high‑quality epidemiological studies. This association was consistent across diverse populations from multiple countries, various sources and metrics of exposure, and a range of study designs.

The peer review process of the NTP systematic review [5] and 2025 meta‑analysis [6] generated many comments on important scientific issues that contributed to the rigor and strength of the final documents. Some of these issues, which were rigorously considered and resolved in the assessments, continue to be raised, and are often highlighted in opinion pieces and the popular press.

Subsequent reviewers of the evidence, especially in regulatory contexts, may encounter similar critiques. This viewpoint aims to assist and inform those tasked with ensuring the safe use of fluoride for oral health.

## Additional File

The additional file for this article can be found as follows:
