# Supplementary material for: Addressing Critiques of the Evidence Linking Fluoride and Children’s IQ
Source: Ann Glob Health. 2025 Dec 12;91(1):83. doi: 10.5334/aogh.4853 (PMC12700148; doi:10.5334/aogh.4853)
Supplement: Supplementary File 1. — Supplemental Material. [file agh-91-1-4853-s1.pdf]

Supplemental Material

<table>
<tr><th>Question of Overlapping Data</th><th>Author Response and Clarification</th></tr>
<tr><td>

Comment

Some commenters have said that Taylor et al. (2025) included overlapping data from multiple publications based on the same Tianjin, China cohort. These include 9 publications: Zhang et al. (2015b), Cui et al. (2018), Yu et al. (2018), Cui et al. (2020), Wang et al. (2020b), Zhao et al. (2021), Wang et al. (2021), Feng et al. (2022), and Xia et al. (2023).

</td><td>

Author Response

Background: Including more than one publication from the same study or cohort in a review is common practice and appropriate when data that would be overlapping are used in separate analyses in a review. The inclusion of overlapping data is inappropriate, and becomes an issue if multiple publications from the same study population are used in the *same analysis*.

Analyses in Taylor et al. (2025) assured and documented that overlapping data from different publications on the same cohort were not used in the same analysis by following the meta-analysis protocol and peer review process. Specifically, the authors ensured that no single analysis included more than one publication from the same underlying study population. That assurance applies to all cohorts including populations in Tianjin, China.

Publications in question:

- Nine publications are cited
    - Four of these publications do have overlapping populations, explained under (A) below. These four publications comprise two distinct sets of overlapping populations and were treated as overlapping in Taylor et al. (2025) to assure that only one publication from an overlapping study was included in any given analysis.
    - The other five of these publications have no evidence of overlapping study populations, explained under (B) below

A. Overlapping study populations recognized and addressed in Taylor et al. (2025)

There are two instances (two publications in each instance) having overlapping study populations:

Overlapping population #1 - Cui et al. (2018) and **Cui et al. (2020)**

- The overlap between these studies were transparently documented and appropriately handled such that the studies were used in separate analyses only as described in the *Supplemental Materials* of Taylor et al. (2025):
    - **Cui et al. (2018)** is only used in the *regression slopes meta-analysis*.
    - **Cui et al. (2020)** is used only in the *mean-effects meta-analysis* and the *mean-effects dose-response meta-analysis*.

</td></tr>
</table>

Question of Overlapping Data	Author Response and Clarification
	Documentation in Taylor et al (2025): The Supplemental Materials in Taylor et al. (2025) note that Cui et al. (2020) is presumably an expanded version of Cui et al. (2018), and thus, the two were not included in the same meta-analysis. **Overlapping population #2 - Yu et al. (2018)** and **Wang et al. (2020b)** - The overlap between these studies were transparently documented and appropriately handled such that the studies were used in separate analyses only as described in the *Supplemental Materials* of *Taylor et al. (2025)*: **Yu et al. (2018)** is used in the three primary meta-analyses: *regression slopes meta-analysis, mean-effects meta-analysis,* and the *mean-effects dose-response meta-analysis* **Wang et al. (2020b)** is used only in a sensitivity analysis for the regression slopes meta-analysis (in which Yu et al. was not used). **Documentation in Taylor et al (2025):** The Supplemental Materials explain: "Yu et al. (2018) and Wang et al. (2020b) used the same study cohort of children recruited in 2015 from rural areas of Tianjin City, China. Since Wang et al. (2020b) (n = 571) used a subset of the original study sample from Yu et al. (2018) (n = 2,886), only results from Yu et al. (2018) were included in the meta-analysis. A sensitivity analysis was performed to evaluate the impact of using the effect estimate from Wang et al. (2020b) rather than the pooled effect estimate from Yu et al. (2018)." **B. Non-overlapping studies (publications with no evidence of overlapping study populations)**<br>There is no evidence to suggest overlapping populations for the remaining five publications cited. Three of these studies were conducted outside of Tianjin City in separate cities and or provinces and therefore no possibility for overlapping populations:<br>**1. Wang et al. (2021)** – Hengshui City<br>**2. Feng et al. (2022)** – Tongxu County, Henan Province<br>**3. Xia et al. (2023)** – Jiangsu Province<br><br>The remaining two publications were both conducted inside Tianjin City (with an estimated population of > 15 million people). However, distinct study details indicate that each publication has a unique study population. Below we present the details of Zhang et al (2015) and Zhao et al (2021) along with Cui et al (2018) and Yu 2018 for additional clarification.<br>**4. Zhang et al. (2015)**<br>**5. Zhao et al. (2021)**

Question of Overlapping Data	Author Response and Clarification					
	**Publication**	**Year(s) of recruitment**	**Ages (grades)**	**Specific location of recruitment**	**Recruitment method**	**Sample size (n)**
	Zhang et al. (2015b)	2011	10–12 years (grade 5)	Two schools in Jinnan district: Gegu Second Primary School and Shuanggang Experimental Primary School	Stratified cluster random sampling based on groundwater fluoride levels	180
	Zhao et al. (2021)	2018	6-12 years (grades 2-5)	Five primary schools selected from five towns	Multistage random and cluster sampling	616
	Yu et al. (2018)	2015	7–13 years	Twenty-four villages (district not reported), not school-based	Multistage random and cluster sampling in rural areas	2,886
	Cui et al. (2018)	2014–2015	7-12 years	Four schools in Jinghai and Dagang districts	Cluster sampling based on water fluoride levels and school cooperation	400

References

Cui Y, Zhang B, Ma J, et al. Dopamine receptor D2 gene polymorphism, urine fluoride, and intelligence impairment of children in China: A school-based cross-sectional study. *Ecotoxicol Environ Saf*. 2018;165:270-277. doi:https://doi.org/10.1016/j.ecoenv.2018.09.018

Cui Y, Yu J, Zhang B, Guo B, Gao T, Liu H. The relationships between thyroid-stimulating hormone and/or dopamine levels in peripheral blood and IQ in children with different urinary iodine concentrations. *Neurosci Lett*. 2020;729:134981. doi:https://doi.org/10.1016/j.neulet.2020.134981

Feng Z, An N, Yu F, et al. Do methylenetetrahydrofolate dehydrogenase, cyclohydrolase, and formyltetrahydrofolate synthetase 1 polymorphisms modify changes in intelligence of school-age children in areas of endemic fluorosis? *Chin Med J*. 2022;135(15):1846-1854. doi:https://doi.org/10.1097/CM9.0000000000002062

Taylor KW, Efrim SE, Sibrizzi CA, Blain RB, Magnuson K, Hartman PA, Rooney AA and Bucher JR, (2025) Fluoride exposure and children's IQ scores: A systematic review and meta-analysis. *JAMA Pediatrs*. 179(3):282-292. doi:10.1001/jamapediatrics.2024.5542

Wang M, Liu L, Li H, et al. Thyroid function, intelligence, and low-moderate fluoride exposure among Chinese school-age children. *Environ Int*. 2020b;134:105229. doi:https://doi.org/10.1016/j.envint.2019.105229

Wang R, He N, Wang Y, Hou G, Zhang P-J. Investigation and analysis of children's dental fluorosis and IQ level in high fluoride district of Hengshui City. *Med Anim Control*. 2021;37(8):796-800.

Xia Y, Xu Y, Shi M, et al. Effects of high-water fluoride exposure on IQ levels in school-age children: A cross-sectional study in Jiangsu, China. *Exposure and Health*. 2023;doi:https://doi.org/10.1007/s12403-023-00597-2

Yu X, Chen J, Li Y, et al. Threshold effects of moderately excessive fluoride exposure on children's health: A potential association between dental fluorosis and loss of excellent intelligence. *Environ Int*. 2018;118:116-124. doi:https://doi.org/10.1016/j.envint.2018.05.042

Zhang S, Zhang X, Liu H, et al. Modifying effect of COMT gene polymorphism and a predictive role for proteomics analysis in children's intelligence in endemic fluorosis area in Tianjin, China. *Toxicol Sci*. 2015b;144:238-245.

Zhao L, Yu C, Lv J, et al. Fluoride exposure, dopamine relative gene polymorphism and intelligence: A cross-sectional study in China. *Ecotoxicol Environ Safety*. Feb 2021;209:111826. doi:https://dx.doi.org/10.1016/j.ecoenv.2020.111826
